# Differences between surviving and non-surviving venous thromboembolism COVID-19 patients: a systematic review

**DOI:** 10.1186/s12959-021-00346-y

**Published:** 2021-12-15

**Authors:** Mauricio Castillo-Perez, Carlos Jerjes-Sanchez, Alejandra Castro-Varela, Jose Gildardo Paredes-Vazquez, Eduardo Vazquez-Garza, Ray Erick Ramos-Cazares, Jose Alfredo Salinas-Casanova, Abigail Montserrat Molina-Rodriguez, Arturo Adrián Martinez-Ibarra, Mario Alejandro Fabiani, Yoezer Z Flores-Sayavedra, Jaime Alberto Guajardo-Lozano, Hector Lopez-de la Garza, Hector Betancourt-del Campo, Daniela Martinez-Magallanes, Jathniel Panneflek

**Affiliations:** 1 Tecnologico de Monterrey. Escuela de Medicina y Ciencias de la Salud., Nuevo Leon San Pedro Garza Garcia, Mexico; 2 Centro de Investigacion Biomedica del Hospital Zambrano Hellion, TecSalud, Escuela de Medicina y Ciencias de la Salud, Tecnologico de Monterrey, Nuevo Leon San Pedro Garza Garcia, Mexico; 3 Instituto de Cardiologia y Medicina Vascular, TecSalud, Escuela de Medicina y Ciencias de la Salud, Tecnologico de Monterrey, Batallón San Patricio 112, Real de San Agustin, Nuevo Leon 66278 San Pedro Garza Garcia, Mexico; 4 Tecnologico de Monterrey, Escuela de Medicina y Ciencias de la Salud, Av. Ignacio Morones Prieto 3000, N.L. CP, 64718 Monterrey, Mexico

**Keywords:** SARS-CoV-2, COVID-19, Venous thromboembolism, Pulmonary embolism, Deep vein thrombosis, Thrombolysis, Anticoagulation

## Abstract

**Background:**

To our knowledge, the treatment, outcome, clinical presentation, risk stratification of patients with venous thromboembolism and COVID-19 have not been well characterized.

**Methods:**

We searched for systematic reviews, cohorts, case series, case reports, editor letters, and venous thromboembolism COVID-19 patients’ abstracts following PRISMA and PROSPERO statements. We analyzed therapeutic approaches and clinical outcomes of venous thromboembolism COVID-19 patients. Inclusion: COVID-19 patients with venous thromboembolism confirmed by an imaging method (venous doppler ultrasound, ventilation-perfusion lung scan, computed tomography pulmonary angiogram, pulmonary angiography). We assessed and reported the original Pulmonary Embolism Severity Index for each pulmonary embolism patient. In addition, we defined major bleedings according to the International Society of Thrombosis and Haemostasis criteria.

**Results:**

We performed a systematic review from August 9 to August 30, 2020. We collected 1,535 papers from PubMed, Scopus, Web of Science, Wiley, and Opengrey. We extracted data from 89 studies that describe 143 patients. Unfractionated and low-molecular-weight heparin was used as parenteral anticoagulation in 85/143 (59%) cases. The Food and Drug Administration-approved alteplase regimen guided the advanced treatment in 39/143 (27%) patients. The mortality was high (21.6%, CI 95% 15.2-29.3). The incidence of major bleeding complications was 1 (0.9%) in the survival group and 1 (3.2%) in the death group. Pulmonary Embolism Severity Index was class I in 11.6% and II in 22.3% in survivors compared to 0% and 6.5% in non-survivors, respectively. Patients who experienced venous thromboembolism events at home were more likely to live than in-hospital events.

**Conclusions:**

We determined a high mortality incidence of pulmonary embolism and a low rate of bleeding. Unfractionated and low-molecular-weight heparin drove parenteral anticoagulation and alteplase the advanced treatment in both groups. The original Pulmonary Embolism Severity Index could be helpful in the risk stratification.

**Supplementary Information:**

The online version contains supplementary material available at 10.1186/s12959-021-00346-y.

## Background

The rapidly evolving coronavirus disease 2019 (COVID-19) global pandemic has been one of the most significant public health challenges since the Spanish flu pandemic over 100 years ago [1]. COVID-19, caused by the severe acute respiratory syndrome coronavirus 2 (SARS-CoV-2), is a multifaceted disease characterized by a wide range of clinical presentations and degrees of severity [2]. In the beginning, the target organ seemed to be only the respiratory system, inducing severe pneumonia and acute respiratory distress syndrome. However, an important lesson learned was that SARS-CoV-2 causes a high prothrombotic state, venous and arterial thrombosis [1]. Thus, the clinical presentation eventually resembles a thrombotic storm characterized by higher D-dimer measurements and high von Willebrand factor levels [3]. Additionally, thrombosis mechanisms linking inflammation pathways, coagulation system activity, immunothrombosis, cytokine storm, and renin-angiotensin-aldosterone system dysregulation [4–8] seem to be involved.

Therefore, in severe COVID-19, venous thromboembolism (VTE) emerges as a critical and frequent complication [9, 10], with a high incidence (15.3%, CI 95% 9.8-21.9) and mortality rate (45.1%, CI 95% 22.0-69.4), in pulmonary embolism (PE) patients [11]. Although there is a trend to better survival in patients treated with heparins (anticoagulation and anti-inflammatory effect) [12, 13], we do not have enough data on the best primary prevention doses, therapeutic approaches, and outcomes [9, 14, 15]. Also, there are no advanced treatment recommendations in massive and submassive PE [16, 17]. Therefore, we performed a systematic review using the Preferred Reporting Items for Systematic Reviews and Metanalyses (PRISMA) statement to determine the therapeutic trends and outcomes in VTE COVID-19 patients. Also, we assessed the original Pulmonary Embolism Severity Index (PESI) in PE patients.

## Methods

### Search strategy

We searched for systematic reviews, cohorts, case series, case reports, editor letters, and VTE COVID-19 patients’ abstracts through the PRISMA statement search [18]. We register the protocol in the International Prospective Register protocol of Systematic Reviews (PROSPERO); registration number: CRD42020203688). The patients must have received anticoagulation or thrombolysis. The objective was to assess the therapeutic trends and clinical outcomes of VTE COVID-19 patients.

Additionally, we analyzed the clinical presentation, risk stratification, and diagnostic approach. We included deep vein thrombosis (DVT) and PE confirmed by an imaging method (venous doppler US, ventilation-perfusion lung scan, computed tomography pulmonary angiogram, pulmonary angiography). We assessed the original PESI since it works better than the simplified PESI [19]. We established two groups, survivors and those who died. We performed a systematic review through PubMed, Scopus, Web of Science, Wiley, and OpenGrey and provided the complete search strategies in the e-Appendix. We used snowballing [20], a manual search to avoid lost reports, controlled vocabulary, and no language restriction. We do not contact authors to obtain additional information in cases with critical missing variables.

### Study selection and data collection

We identified potentially eligible studies by examining titles and abstracts. We obtained full papers to assess eligibility criteria before the critical appraisal and extracted cases that met the eligibility criteria. All investigators analyzed data extraction of every case report to improve quality data extraction. The corresponding author is a cardiologist with expertise in the field (CJS). We conducted a group discussion daily to assess all the information extracted from the cases included in a database. Disagreements were solved posteriorly by consensus. We performed two meetings to ensure the data’s quality through a random review of 20% of the papers. The primary outcomes were therapeutic approaches, in-hospital death, intracranial hemorrhage (ICH), major, and minor.

Additionally, we analyzed the clinical presentation, the PE risk, COVID-19 severity, VTE primary prevention, and the thrombus’s location in the pulmonary circulation. According to the International Society of Thrombosis and Haemostasis criteria, we defined major bleedings [21]; we established the presence of right ventricular dysfunction according to the European Society of Cardiology guidelines of PE: right ventricular end-diastolic diameter/left ventricular end-diastolic diameter ratio ≥2:1, (b) regional or global right ventricular hypokinesis, (c) McConnell’s sign, (d) right ventricular diameter >35 mm, (e) systolic pulmonary arterial pressure ≥50 mm Hg; B-type brain natriuretic peptide (BNP) measurement (>90 pg/mL) or N-terminal proBNP (NT-proBNP) (>300 pg/mL); dynamic electrocardiographic changes (new complete or incomplete right bundle-branch block, anteroseptal ST elevation or depression, or anteroseptal T-wave inversion) [22]; other definitions, including the PESI score, massive PE and intensive care unit (ICU) VTE risk factors, are available in the e-Appendix.

Based on the high SARS-CoV-2 thrombogenicity and to understand its behavior in the venous system, we also analyzed acute cerebral venous sinus thrombosis (CVST), whether associated or not with VTE.

### Statistical analysis

We used summary statistics for continuous and categorical variables according to their types and distributions. We report the frequency and percentage (n >20) for categorical variables, and for continuous variables, we report the mean and standard deviation. We used the IBM SPSS® software platform for descriptive statistical analysis.

## Results

**Fig. 1:**
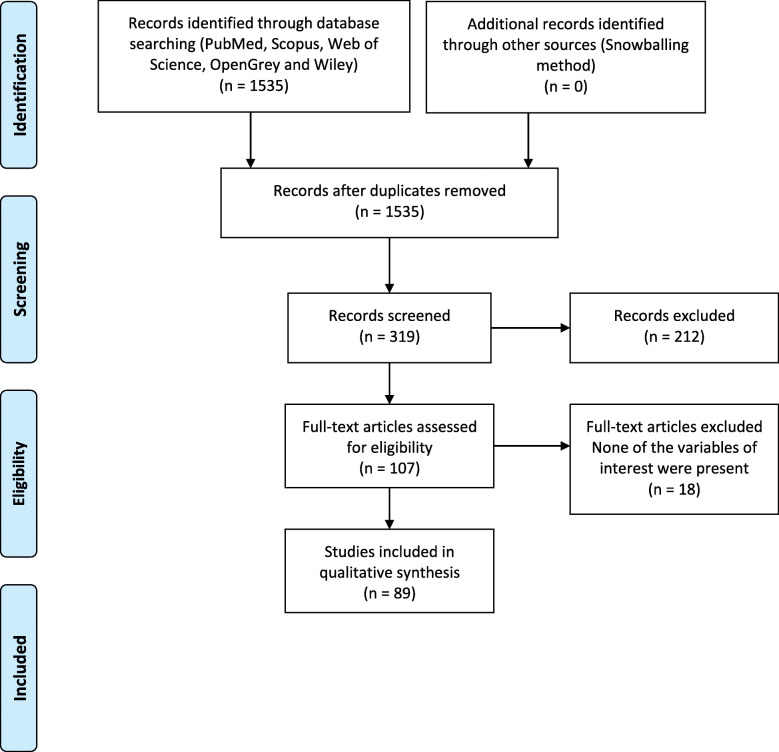
PRISMA flow diagram

We carried out the systematic review from August 9 to August 30, 2020. Figure 1 shows the flowchart, including the four phases of PRISMA, and we obtained, eliminated, and excluded duplicated reports. In the identification phase, we collected 1,535 papers from PubMed, Scopus, Web of Science, Wiley, and Opengrey. Next, we carefully reviewed the full text for eligibility criteria and selected 107 reports for the quality assessment. Finally, we extracted the data for this review from 89 studies (references in supplementary material).

### Baseline demographics and primary outcomes

Table 1 shows baseline demographics, clinical presentation, VTE and PE risk factors, DVT classification, PESI, and VTE onset. We identified 143 COVID-19 patients with VTE; most were relatively young overweight males with isolated PE with or without proximal DVT. The earliest clinical PE findings were severe oxygen desaturation, sudden dyspnea, and leg pain in DVT survived patients (Table 1). A remarkable characteristic was the lowest oxygen saturation in those who died. Among the usual comorbidities in COVID-19, hypertension had a higher incidence in patients who died. Cardiovascular risk factors (hypertension and diabetes) and those associated with in-hospital and ICU stay were more prevalent in those who died (Table 1). The proportion of low-risk and submassive PE was higher in patients who survived than those who died, where massive PE was predominant (Table 1). In this group, the detection of proximal or distal DVT was scarce. The original PESI classes II and III identified patients who lived (Table 1). Finally, patients with acute VTE events at home were more likely to live than in-hospital events. We identified reduced thromboprophylaxis use in both groups (Table 2). Initial treatment shows that unfractionated and low-molecular-weight heparin drove parenteral anticoagulation in both groups. Also, direct-acting oral anticoagulant use was rare. Alteplase 100 mg 2-hours infusion was the advanced treatment in both groups (Table 2). The mortality was high (21.6%, CI 95% 15.2-29.3), and there was a low incidence of bleeding complications, including ICH, in those who survived (Table 2).

**Table 1:** Baseline demographics, clinical presentation, VTE risk factors, PE risk stratification, VTE classification, DVT classification, PESI, and VTE onset

Variables		All patients *N* = 143 (%)		Survival *N* = 112 (%)		Death *N* = 31 (%)
Age (years), mean ± SD		58.5 ± 12.7		58.1 ± 13.6		60.0 ± 8.8
Gender (male)		91 (63.6)		70 (62.5)		21 (67.7)
BMI (kg/m2), mean ± SD		30.9 ± 5.6		30.3 ± 5.6		31.6 ± 5.6
**VTE Clinical presentation**						
O2 saturation (%), mean ± SD		87.9 ± 7.6		88.3 ± 7.4		85 ± 9.4
Sudden dyspnea		31 (21.7)		29 (25.9)		2 (6.5)
Progressive dyspnea		26 (18.2)		23 (20.5)		3 (9.7)
Pleuritic chest pain		20 (14)		19 (17)		1 (3.2)
Ischemic chest pain		2 (1.4)		2 (1.8)		0 (0)
Leg pain		13 (9.1)		11 (9.8)		2 (6.5)
**Medical history and risk factors**						
Hypertension		50 (35)		34 (30.4)		16 (51.6)
Diabetes		33 (23.1)		25 (22.3)		8 (25.8)
Lung disease		16 (11.2)		15 (13.4)		1 (3.2)
Medical history of cancer		7 (4.9)		5 (4.5)		2 (6.5)
Active cancer		5 (3.5)		3 (2.7)		2 (6.5)
Previous venous thromboembolism		2 (1.4)		2 (1.8)		0 (0)
**In-hospital and ICU risk factors**						
Immobilization		88 (61.5)		63 (56.3)		25 (80.6)
Sedation		51 (35.7)		27 (24.1)		24 (77.4)
Central venous lines		52 (36.4)		28 (25)		24 (77.4)
Vasopressors		15 (10.5)		4 (3.6)		11 (35.5)
**PE risk stratification (ACC/AHA)**						
Low risk		24 (18.3)		23 (20.5)		1 (3.2)
Submassive		31 (23.7)		29 (25.9)		2 (6.5)
Massive		39 (29.8)		21 (18.8)		18 (58)
Unable to classify		37 (28.2)		29 (25.9)		8 (25.8)
**VTE classification**						
Isolated pulmonary embolism		112 (78.3)		85 (75.9)		27 (87.1)
Isolated deep venous thrombosis		12 (8.4)		10 (8.9)		2 (6.5)
Pulmonary embolism plus DVT		18 (12.6)		16 (14.3)		2 (6.5)
Pulmonary embolism plus CVT		1 (0.7)		1 (0.9)		0 (0)
**DVT classification**						
Proximal DVT		14 (9.8)		11 (9.8)		3 (9.7)
Distal DVT		5 (3.5)		5 (4.5)		0 (0)
Proximal plus distal DVT		5 (3.5)		5 (4.5)		0 (0)
Upper limb DVT		6 (4.2)		4 (3.6)		2 (6.5)
**Original PESI**						
I (Very low risk)		13 (9.1)		13 (11.6)		0 (0)
II (Low risk)		27 (18.9)		25 (22.3)		2 (6.5)
III (Intermediate risk)	45 (31.5)		37 (33)		8 (25.8)	
IV (High risk)		11 (7.7)		11 (9.8)		0 (0)
V (Very high risk)		35 (24.5)		19 (17)		16 (51.6)
**VTE onset**						
Home		53 (37.1)		48 (42.9)		5 (16.1)
In-hospital		90 (62.9)		64 (57.1)		26 (83.9)

*BMI* body mass index, *ICU* intensive care unit, *PESI* Pulmonary Embolism Severity Index, *VTE* venous thromboembolism, *DVT* deep venous thrombosis, *CVT* cerebral venous thrombosis, *PE* pulmonary embolism, *ACC/AHA* American College of Cardiology/American Heart Association

**Table 2:** Therapeutic approaches and outcomes

Variables		All patients *N* = 143 (%)		Survival *N* = 112 (%)		Death *N* = 31 (%)
**Thromboprophylaxis**						
Unfractionated heparin	17 (11.9)		11 (9.8)		6 (19.4)	
Low-molecular weight heparin	31 (21.7)		20 (17.9)		11 (35.5)	
Unspecified	3 (2.1)		2 (1.8)		1 (3.2)	
Not received	89 (62.2)		77 (68.8)		12 (38.7)	
**Treatment**						
Unfractionated heparin		28 (19.6)		22 (19.6)		6 (19.4)
Low-molecular-weight-heparin		57 (39.9)		49 (43.8)		8 (25.8)
Warfarin		0 (0)		0 (0)		0 (0)
Fondaparinoux		3 (2.1)		3 (2.7)		0 (0)
Direct-acting oral anticoagulants		8 (5.6)		8 (7.1)		0 (0)
Apixaban		4 (2.8)		4 (3.6)		0 (0)
Rivaroxaban		2 (1.4)		2 (1.8)		0 (0)
Unspecified DOACs		2 (1.4)		2 (1.8)		0 (0)
Alteplase 100 mg		33 (23.1)		20 (17.9)		13 (41.9)
Alteplase 50 mg		6 (4.2)		4 (3.6)		2 (6.5)
Tenecteplase		1 (0.7)		0 (0)		1 (3.2)
Catheter-directed thrombolysis		1 (0.7)		1 (0.9)		0 (0)
Ultrasound-facilitated catheter-directed thrombolysis		3 (2.1)		3 (2.7)		0 (0)
Mechanical thrombectomy		3 (2.1)		3 (2.7)		0 (0)
Surgical thrombectomy		4 (2.8)		3 (2.7)		1 (3.2)
**Outcomes**						
Death		31 (21.6)		112 (78.39)		31 (21.6)
Intracranial hemorrhage		2 (1.4)		1 (0.9)		1 (3.2)
Major bleeding		2 (1.4)		1 (0.9)		1 (3.2)
Minor bleeding		2 (1.4)		1 (0.9)		1 (3.2)

*DOACs* direct-acting oral anticoagulants

Table 3 shows characteristics of VTE, including biomarkers, imaging studies, and severity, laboratories. Imagin and previous anticoagulation use related to COVID-19. Patients who died had a higher D dimer expression and right ventricular dysfunction (Table 3), and the use of biomarkers was low. [22] (Table 3). The computed tomography pulmonary angiography (CTPA) demonstrated a wide distribution of thrombus locations in surviving patients (Table 3). The variables mainly related to mortality were acute respiratory distress syndrome, mechanical ventilation, ICU stay, and higher C reactive protein measurements in patients with PE associated with severe COCID-19 patients.

**Table 3:** Characteristics of venous thromboembolism in COVID-19 patients

Variables	All patients *N* = 143 (%)	Survival *N* = 112 (%)	Death *N* = 31 (%)
**Biomarkers**			
D-dimer (mcg/mL), median (IQR)	7794 (3320 – 17,460)	7700 (3200 – 16,125)	8897 (4352 – 33,175)
Hs-cTn (ng/mL), median (IQR)	57 (14.5 – 191)	-	-
Ferritin (ng/mL), median (IQR)	765 (402 – 1456)	-	-
**Imaging studies**			
Right ventricular dysfunction (TTE)	56 (39.2)	35 (31.3)	21 (67.8)
**CTPA**			
Saddle PE	10 (7)	9 (8)	1 (3.2)
Main branches	38 (26.6)	34 (30.4)	4 (12.9)
Lobar branches	22 (15.4)	19 (17)	3 (9.7)
Segmental branches	27 (18.9)	23 (20.5)	4 (12.9)
Subsegmental branches	7 (4.9)	7 (6.3)	0 (0)
Doppler US and DVT	30 (20.9)	25 (22.3)	5 (16.1)
**COVID-19 severity**			
Asymptomatic	14 (9.8)	12 (10.7)	2 (6.5)
Mild symptoms	9 (6.3)	8 (7.1)	1 (3.2)
Fever	27 (18.9)	23 (20.5)	4 (12.9)
Pneumonia	62 (43.4)	55 (49.1)	7 (22.6)
ARDS	58 (40.6)	37 (33)	21 (67.7)
Mechanical ventilation	56 (39.2)	32 (28.6)	24 (77.4)
ICU	69 (48.3)	40 (35.7)	29 (93.5)
**Laboratories**			
Leukocytes (10(9) u/L), median (IQR)	11.9 (9.7 – 15.4)	11.4 (9.4 – 13.6)	13.8 (10.8 – 20.3)
Lymphocytes (10(3) u/L), mean ± SD	928.3 ± 448.5	994.1 ± 461.5	731.1 ± 360.1
Platelets (10(3) u/L), mean ± SD	246.8 ± 129.8	254.4 ± 122.9	233.7 ± 143.7
LDH (U/L), median (IQR)	575 (391.8 – 739.3)	-	-
CRP (mg/L), median (IQR)	113.1 (50.6 – 222.5)	92.9 (50 – 160)	244.9 (154 – 345.4)
RT-PCR SARS-CoV-2 (+)	142 (99.3)	111 (99.1)	31 (100)
**Imagen studies**			
Bilateral infiltrates (chest X-ray)	49 (34.3)	37 (33)	12 (38.7)
CT with CO-RADS 5	49 (34.3)	43 (38.4)	6 (19.4)
**Previous anticoagulation treatment**			
Direct-acting oral anticoagulants	1 (0.7)	1 (0.9)	0 (0)
Vitamin K antagonists	2 (1.4)	1 (0.9)	1 (3.2)

*TTE* transthoracic echocardiogram, *CTPA* computed tomographic pulmonary angiography, *PE* pulmonary embolism, *US* ultrasound, *DVT* deep vein thrombosis, *ARDS* acute respiratory distress syndrome, *ICU* intensive care unit, *LDH* lactate dehydrogenase, *CRP* C-reactive protein, *RT-PCR* reverse transcription-polymerase chain reaction, *CO-RADS 5* COVID-19 Reporting and Data System with typical imaging for COVID-19

### Cerebral venous sinus thrombosis

We identified 15 young patients with a similar gender proportion practically without a history of contraceptives (Table 4). CVST clinical presentation included neurologic alterations at home, abnormal D dimer measurements, and only one case associated with a submassive PE. Most patients were asymptomatic or had COVID-19 pneumonia. Despite in-hospital primary prevention, five patients had CVST. We identified a remarkably high prevalence of ICH (10/15 patients) (66.7% CI95 38.4-88.2) and increased mortality (3/15 patients) (20% CI954.3-48.1) (Table 4).

**Table 4:** Cerebral venous sinus thrombosis

Variables	*N* = 15
Age	56 ± 14.3
Gender (male)	7
Risk factors	
Comorbidities (≥1)	2
Oral contraceptives	2
D-dimer (mcg/mL), mean ± SD	3698.4 ± 2017.3
Submassive pulmonary embolism	1
**CVT presentation**	
Altered mental status	6
Headache	8
Aphasia	6
Hemiparesis	7
Seizures	4
At home	9
**COVID-19 clinical presentation**	
Fever	3
Progressive dyspnea	3
Asymptomatic	3
Mild symptoms	1
Pneumonia	6
Computed tomography with CO-RADS 5	6
Thromboprophylaxis	5
**Treatment and outcomes**	
Unfractionated heparin	3
Low-molecular-weight-heparin	12
Intracranial hemorrhage	7
Death	3

*CO-RADS 5* COVID-19 Reporting and Data System with typical imaging for COVID-19

## Discussion

This systematic review highlights the therapeutic trends and outcomes of VTE survivors compared with those who died. The main observations were: First, unfractionated and low-molecular-weight heparin was the cornerstone in the VTE treatment. Also, 2-hours alteplase infusion was the most frequent advanced treatment in PE patients. Second, we identified high mortality in the ICU associated with severe COVID-19 with a low incidence of bleeding complications in massive PE. Third, the original PESI score II-III recognized patients who survived, suggesting its usefulness in the risk stratification in COVID-19 patients. Fourth, elevated C reactive protein and D dimer measurements and right ventricular dysfunction identified poor in-hospital outcomes. Finally, the exploratory analysis showed the same high ICH incidence in CVST mild COVID-19 patients than non-COVID-19 patients [23].

Recent systematic reviews and meta-analyses focused on the incidence, primary and secondary VTE prevention, bleeding complications [24–27], and the association of D-dimer with mortality [28, 29]. Therefore, therapeutic approaches, outcomes, clinical presentation, risk stratification, and patient characteristics are unclear.

Although still under debate, recent evidence from a small sample suggests that patients with severe COVID-19 disease are at high risk for thromboinflammation since they have SARS-CoV-2 infection, risk factors, cardiovascular, renal, or chronic pulmonary inflammatory comorbidities [2]. An increased frequency of arterial and venous thrombosis at the beginning of the pandemic was remarkable [30]. VTE is now recognized as among the predominant cardiovascular hazards [30], with the highest incidence in the intensive care unit setting (25%), increasing to 69% after surveillance venous ultrasonography [30]. Also, thromboprophylaxis, the foundation to prevent in-hospital VTE, fails in a subset of COVID-19 patients [30]. Additionally, quantifying the risk of thrombosis and cardiovascular complications is complicated in this heterogeneous population by reports of limited sample size, restriction of assessments to the ICU setting, outcome definitions, and differing thromboprophylaxis strategies [30].

Our findings suggest that intravenous or subcutaneous anticoagulation remains the cornerstone of therapy in deep venous thrombosis and PE COVID-19 patients. Strategies for reperfusion therapy included the thrombolysis regimen recommended for international guidelines [22] or “safe dose” in PE patients [31–33]. The rationale for advanced treatment in PE is to avert or improve impending clinical instability secondary to right ventricular dysfunction to improve the outcome. The presence of several pulmonary hypertension mechanisms (PE, hypoxic vasoconstriction, pulmonary microthrombi, ACE2 dysregulation, and cytokine storm) inducing right ventricular dysfunction suggests the possibility to obtain a CTPA before clinical decision-making in this population [34]. In the presence of high clinical suspicion and clinical instability, systemic thrombolysis use has evidence level IC [22]. Despite systemic thrombolysis, bleeding complication incidence was lower (0.9% vs. 3.2%) than recent evidence (21.4%) using intermediate- or full-heparin dose without advanced treatment and bleeding definitions according to the individual studies [27]. This difference in the incidence of bleeding complications is unclear because relevant clinical or significant bleedings are usually reported. We showed high mortality (46% in massive PE in severe COVID-19 patients. It is higher than observed in massive PE non-COVID-19 patients (33%) [35]; the mortality rates observed are also related to severe COVID-19 and higher than previous other viral pandemics experienced in the past [36]. Additionally, mortality appears to be multifactorial and driven by adult respiratory distress syndrome (ARDS) and massive PE. In the absence of a validated risk score for patients with severe COVID-19 and PE, current risk stratification in PE [22] could lose accuracy and explain the high percentage of unclassified PE patients.

The original PESI score is a helpful tool for immediate and bedside risk stratification [22]; if this score helps to stratify bedside high clinical suspicion PE in COVID-19 patients is unanswered. The original PESI risk score had greater precision in identifying low and intermediate PE risks and identified a high proportion of high-risk patients with very high risk [19]. In addition, COVID-19 in the health systems usually conditions a delay recommended diagnostic approaches in high clinical suspicion PE patients [22]; thus, the original PESI score could be helpful in high clinical suspicion COVID-19 patients. However, clinicians should also consider that the simplified PESI score may fail [37], and a multimodal approach improves risk stratification accuracy. (PESI score definition is available in the e-Appendix).

Another remarkable finding shows VTE events despite thromboprophylaxis. Recent evidence indicates that thrombotic events occur primarily within the first ten days after admission [38]. In addition, Hardy et al. [39] observed an increase in thrombin generation associated with a decrease in overall fibrinolytic capacity during the first week of hospitalization, resulting in a strong procoagulant state. Thus, current evidence suggests administering heparin at standard doses in non-critically ill patients without risk factors for thrombosis or at a high dose for critically ill patients (intermediate or therapeutic dose) [40].

Additionally, high-dose thromboprophylaxis might be adjusted according to inflammation’s progression without increasing bleeding Risk in critically ill COVID-19 patients [38]. Randomized controlled trials comparing different thromboprophylaxis doses are needed to establish the best therapeutic approach [38]. The most consistent biomarker abnormalities related to mortality were higher C-reactive protein and D-dimer measurement levels, both associated with ICU admission and death [15]. Additionally, several plausible reasons for elevated D-dimer in patients with SARS-CoV-2: severe infection, VTE, pulmonary and coronary microthrombus, acute kidney, cardiac injury, and pro-inflammatory cytokines [29].

Overlapping severe COVID-19 pneumonia and PE is a challenge, and any pneumonia increases VTE risk [34, 41, 42]. A higher D-dimer measurement and severe oxygen desaturation are possible clinical markers to establish high clinical suspicion and PE severity. Recently, in a case series, the clinical presentation was similar: persistent or worsening respiratory symptoms increased oxygen requirements and DD levels several-fold higher [43]. We suggest that physicians in charge consider these clinical variables and never ignore abnormal or significantly elevated D-dimer because it is an expression of the coagulation system and secondary fibrinolysis activity, suggesting a high risk of acute thrombosis [34]. Sudden hypotension could be another clinical element for PE suspicion in the setting of pneumonia COVID-19 [34]. In the group with CVST, only two patients had a history of oral contraceptives and no history of hereditary prothrombotic factors. These findings suggest an essential role of SARS-CoV-2 in pathogenicity as a trigger of thrombosis. Although early ICH (present at the time of diagnosis) is a frequent complication (40%) [44, 45], current evidence demonstrates a low incidence of new ICH after initiating treatment with anticoagulation [23, 44–46]. Our findings identified a high ICH incidence, probably secondary to CVST. Although anticoagulation is the standard of care in CVST patients (avoid thrombus growth, prevent VTE), the high prevalence of ICH suggests that physicians in charge have to be warning for early detection of this feared complication [45].

### Study limitations

The significant limitations of the study included a potential loss of case reports from search engines. There is a trend not to report patients with poor in-hospital outcomes or serious adverse events. In addition, it was not possible to obtain information on the timing of the D-dimer measurements and other biomarkers and bleeding complications outcome in the follow-up. We got the most information from case reports, and we did not contact any author. Additionally, the results should be analyzed with caution as most papers are case reports or case series. Despite a large number of published studies in Covid VTE, the number of studies that report outcomes based on treatments is unacceptably small to draw new conclusions, given the different stages of the pandemic, Covid-19 treatments, and international differences. The usable studies had in common and why the other studies were rejected; could this be the basis of reporting standards for the pandemic to help a unified assessment. The impact of VTE on critically ill patients seems no different from other diseases - so is it just that we cannot cure the underlying disease, or is there something unique about COVID-19 thrombosis.

## Conclusions

This systematic review analyzes 143 survivors and non-survivors VTE COVI-19 patients. We determined a high mortality incidence of pulmonary embolism (21.6%) and a low rate of bleeding. Unfractionated and low-molecular-weight heparin drove parenteral anticoagulation and alteplase the advanced treatment in both groups. The original PESI could be helpful in risk stratification. However, the minuscule number of evaluated patients cannot possibly be representative, and therefore, the international community should urgently agree on reporting standards to answer the remaining questions in Covid-19. Prospective clinical trials are mandatory to elucidate the optimal primary or secondary prevention and advanced treatment in this population of patients.

## Supplementary Information

**Additional file 1.**

## Data Availability

Not applicable.

## Abbreviations

COVID-19: Coronavirus disease 2019
SARS-CoV-2: Severe acute respiratory syndrome coronavirus 2
VTE: Venous thromboembolism
PE: Pulmonary embolism
PESI: Original Pulmonary Embolism Severity Index
PROSPERO: The International Prospective Register protocol of Systematic Reviews
PRISMA: The Preferred Reporting Items for Systematic Reviews and Metanalyses
DVT: Deep vein thrombosis
PESI: Simplified Pulmonary Embolism Severity Index
BNP: B-type brain natriuretic peptide
NT-proBNP: N-terminal proBNP
CVT: Cerebral vein thrombosis
ICU: Intensive care unit
ICH: Intracranial hemorrhage
CTPA: Computed tomography pulmonary angiography
