# Supplementary material for: Differences between surviving and non-surviving venous thromboembolism COVID-19 patients: a systematic review
Source: Thromb J. 2021 Dec 15;19:101. doi: 10.1186/s12959-021-00346-y (PMC8672331; doi:10.1186/s12959-021-00346-y)
Supplement: Supplementary file 1 — Additional file 1. [file 12959_2021_346_MOESM1_ESM.docx]

**e-Appendix**

**Search strategy**

(("thrombosis"[MeSH Terms] OR "thrombosis"[All Fields]) OR ("venous thromboembolism"[MeSH Terms] OR ("venous"[All Fields] AND "thromboembolism"[All Fields]) OR "venous thromboembolism"[All Fields]) OR ("pulmonary embolism"[MeSH Terms] OR ("pulmonary"[All Fields] AND "embolism"[All Fields]) OR "pulmonary embolism"[All Fields]) OR ("venous thrombosis"[MeSH Terms] OR ("venous"[All Fields] AND "thrombosis"[All Fields]) OR "venous thrombosis"[All Fields] OR ("deep"[All Fields] AND "vein"[All Fields] AND "thrombosis"[All Fields]) OR "deep vein thrombosis"[All Fields]) OR ("disseminated intravascular coagulation"[MeSH Terms] OR ("disseminated"[All Fields] AND "intravascular"[All Fields] AND "coagulation"[All Fields]) OR "disseminated intravascular coagulation"[All Fields] OR ("intravascular"[All Fields] AND "disseminated"[All Fields] AND "coagulation"[All Fields]) OR "intravascular disseminated coagulation"[All Fields]) OR thrombolysis[All Fields] OR anticoagulation[All Fields] OR heparin[All Fields] OR enoxaparin[All Fields] OR cor pulmonale[All Fields]) AND (COVID[All Fields] OR ("severe acute respiratory syndrome coronavirus 2"[Supplementary Concept] OR "severe acute respiratory syndrome coronavirus 2"[All Fields] OR "sars cov 2"[All Fields]))

**Definitions**

- SARS-CoV-2: severe acute respiratory syndrome coronavirus 2

- COVID-19: coronavirus disease 2019

- Venous thromboembolism: encompasses deep vein thrombosis and its most dangerous complication, acute pulmonary embolism, and is globally the third most frequent acute cardiovascular syndrome[1].

- Deep vein thrombosis: proved by venous US[2].

- Proximal DVT: located in the popliteal, femoral or iliac veins

- Distal DVT: no proximal component, is located below the knee, and is confined to the calf veins (peroneal, posterior, anterior tibial, and muscular veins).

- Pulmonary embolism: proved by V/Q lung scan diagnosis, CT angiography, pulmonary angiography or transesophageal echocardiogram. Also, we considered PE in those with high-clinical suspicion, in-transit thrombus, and right ventricular dysfunction evidenced by echocardiogram with or without clinical instability[3].

- Massive pulmonary embolism: sustained hypotension systolic blood pressure <90 mmHg for at least 15 minutes or requiring inotropic support, not due to a cause other than PE, such as arrhythmia, hypovolemia, sepsis, or left ventricular dysfunction, pulselessness, or persistent profound bradycardia (heart rate 40 bpm with signs or symptoms of shock.

- Submassive pulmonary embolism: systolic blood pressure >90 mmHg, but with either right ventricular dysfunction or myocardial necrosis[3].

- Low-risk pulmonary embolism: systolic blood pressure >90 mmHg without right ventricular dysfunction and biomarkers expression.

- Right ventricular dysfunction: on echocardiography or angio CT: right ventricular end diastolic diameter/left ventricular end diastolic diameter ratio (RVEDD/LVEDD) >2:1, (b) regional or global right ventricular hypokinesis, (c) McConnell´s sign, (d) right ventricular diameter >35 mm, (e) systolic pulmonary arterial pressure >50 mm Hg; BNP measurement (>90 pg/mL) or N-terminal proBNP (NT-proBNP) (>300 pg/mL)^2^; dynamic electrocardiographic changes (new complete or incomplete right bundle-branch block, anteroseptal ST elevation or depression, or anteroseptal T-wave inversion)^2^.

- Isolated or multiple segmental or subsegmental thrombi on CTA[4].

- VTE strong risk factors (OR>10): fracture of the lower limb, hospitalization for heart failure or AF/flutter (within the previous three months), hip or knee replacement, major trauma, myocardial infarction (within last three months), previous VTE, spinal cord injury[1].

- VTE moderate risk factors (OR 2-9): arthroscopic knee surgery, autoimmune diseases, blood transfusion, central venous lines, IV catheters, and leads, chemotherapy, HF, erythropoiesis-stimulating agents, hormone replacement therapy, in vitro fertilization, oral contraceptive therapy, post-partum period, infection, inflammatory bowel disease, cancer, paralytic stroke, superficial vein thrombosis, thrombophilia[1].

- VTE weak risk factors (OR<2): bed rest >3 days, DM, HT, immobility due to sitting, increasing age, laparoscopic surgery, obesity, pregnancy, varicose veins[1].

- ICU VTE risk factors: sedation, immobilization, vasopressors, or central venous catheters[5].

- PESI score: predicts 30-day outcome of patients with pulmonary embolism using 11 clinical criteria: age, sex, history of cancer, history of heart failure, history of chronic lung disease, heart rate ≥110, systolic BP <100 mmHg, respiratory rate ≥30, temperature <36ºC/96.8ºF, altered mental status and O2 saturation <90%[6].

- sPESI score: predicts 30-day outcome of patients with pulmonary embolism, with fewer criteria than the original PESI: age ≥80, history of cancer, history of chronic cardiopulmonary disease, heart rate ≥110, systolic BP ≥100 mmHg, and O2 saturation ≥90%[6].

- In-transit thrombi: blood thrombus located inside right cavities of the heart, classified into three groups: type A (the most frequent, highly mobile, with serpent form); type B (mostly nonmobile, with a left heart thrombus form); type C (highly mobile, can remember a myxoma)[7].

- Immunothrombosis: thrombosis mechanism that includes innate immune mechanisms, the neutrophil extracellular genetic traps, and the immunothrombosis dysregulation[8].

- Cytokine storm: Release of inflammatory cytokines triggered by a wide variety of molecular patterns that can be found in microorganisms, which will induce new cytokine production and release that will, in turn, cause cell and organ damage. This cytokine storm is likely responsible for the diverse, local, and remote, signs associated with infection[9].

1. Konstantinides SV, Meyer G, Becattini C, et al (2019) 2019 ESC Guidelines for the diagnosis and management of acute pulmonary embolism developed in collaboration with the European Respiratory Society (ERS). European Heart Journal ehz405. https://doi.org/10.1093/eurheartj/ehz405

2. Mazzolai L, Aboyans V, Ageno W, et al (2018) Diagnosis and management of acute deep vein thrombosis: a joint consensus document from the European Society of Cardiology working groups of aorta and peripheral vascular diseases and pulmonary circulation and right ventricular function. European Heart Journal 39:4208–4218. https://doi.org/10.1093/eurheartj/ehx003

3. Jaff MR, McMurtry MS, Archer SL, et al (2011) Management of Massive and Submassive Pulmonary Embolism, Iliofemoral Deep Vein Thrombosis, and Chronic Thromboembolic Pulmonary Hypertension: A Scientific Statement From the American Heart Association. Circulation 123:1788–1830. https://doi.org/10.1161/CIR.0b013e318214914f

4. ten Cate V, Eggebrecht L, Schulz A, et al (2020) Isolated Pulmonary Embolism Is Associated With a High Risk of Arterial Thrombotic Disease. Chest 158:341–349. https://doi.org/10.1016/j.chest.2020.01.055

5. Kollias A, Kyriakoulis KG, Dimakakos E, et al (2020) Thromboembolic risk and anticoagulant therapy in COVID‐19 patients: emerging evidence and call for action. Br J Haematol bjh.16727. https://doi.org/10.1111/bjh.16727

6. Jiménez D (2010) Simplification of the Pulmonary Embolism Severity Index for Prognostication in Patients With Acute Symptomatic Pulmonary Embolism. Arch Intern Med 170:1383. https://doi.org/10.1001/archinternmed.2010.199

7. Jerjes-Sánchez C Current Guidelines and Recommendations for Thrombolysis Use in Pulmonary Embolism. In: Thrombolysis in Pulmonary Embolism. Springer International Publishing, Switzerland, pp 107–130

8. Vazquez-Garza E, Jerjes-Sanchez C, Navarrete A, et al (2017) Venous thromboembolism: thrombosis, inflammation, and immunothrombosis for clinicians. J Thromb Thrombolysis 44:377–385. https://doi.org/10.1007/s11239-017-1528-7

9. Chousterman BG, Swirski FK, Weber GF (2017) Cytokine storm and sepsis disease pathogenesis. Semin Immunopathol 39:517–528. https://doi.org/10.1007/s00281-017-0639-8

**Studies included:**

1. Audo A, Bonato V, Cavozza C, Maj G, Pistis G, Secco GG. Acute Pulmonary Embolism in SARS-CoV-2 Infection Treated with Surgical Embolectomy. The Annals of Thoracic Surgery. 2020 Apr;S0003497520306160.

2. Le Berre A, Marteau V, Emmerich J, Zins M. Concomitant acute aortic thrombosis and pulmonary embolism complicating COVID-19 pneumonia. Diagnostic and Interventional Imaging. 2020 May;101(5):321–2.

3. Bomhof G, Mutsaers PGNJ, Leebeek FWG, Boekhorst PAW, Hofland J, Croles FN, et al. COVID‐19‐associated immune thrombocytopenia. Br J Haematol [Internet]. 2020 Jul [cited 2020 Aug 31];190(2). Available from: https://onlinelibrary.wiley.com/doi/abs/10.1111/bjh.16850

4. Creel-Bulos C, Hockstein M, Amin N, Melhem S, Truong A, Sharifpour M. Acute Cor Pulmonale in Critically Ill Patients with Covid-19. N Engl J Med. 2020 May 6;e70.

5. Danzi GB, Loffi M, Galeazzi G, Gherbesi E. Acute pulmonary embolism and COVID-19 pneumonia: a random association? European Heart Journal. 2020 Mar 30;ehaa254.

6. Fabre O, Rebet O, Carjaliu I, Radutoiu M, Gautier L, Hysi I. Severe Acute Proximal Pulmonary Embolism and COVID-19: A Word of Caution. The Annals of Thoracic Surgery. 2020 Apr;S0003497520305701.

7. Foch E, Allou N, Vitry T, Masse L, Allyn J, Andre M, et al. Pulmonary embolism in a returning traveller with COVID-19 pneumonia. Journal of Travel Medicine. 2020 Aug 20;27(5):taaa063.

8. Garaci F, Di Giuliano F, Picchi E, Da Ros V, Floris R. Venous cerebral thrombosis in COVID-19 patient. Journal of the Neurological Sciences. 2020 Jul;414:116871.

9. Griffin DO, Jensen A, Khan M, Chin J, Chin K, Saad J, et al. Pulmonary Embolism and Increased Levels of d -Dimer in Patients with Coronavirus Disease. Emerg Infect Dis [Internet]. 2020 Aug [cited 2020 May 1];26(8). Available from: http://wwwnc.cdc.gov/eid/article/26/8/20-1477_article.htm

10. Harsch IA, Skiba M, Konturek PC. SARS-CoV-2 pneumonia and pulmonary embolism in a 66-year-old female. Polish Archives of Internal Medicine [Internet]. 2020 May 1 [cited 2020 Aug 31]; Available from: https://www.mp.pl/paim/issue/article/15333

11. Horowitz JM, Yuriditsky E, Henderson IJ, Stachel MW, Kwok B, Saric M. Clot in Transit on Transesophageal Echocardiography in a Prone Patient with COVID-19 Acute Respiratory Distress Syndrome. CASE. 2020 Aug;4(4):200–3.

12. Ioan A-M, Durante-López A, Martínez-Milla J, Pérez-Calvo C, Santos A. Pulmonary embolism in COVID-19. When nothing is what it seems. Revista Española de Cardiología (English Edition). 2020 Aug;73(8):665–7.

13. Jafari R, Cegolon L, Jafari A, Kashaki M, Otoukesh B, Ghahderijani BH, et al. Large saddle pulmonary embolism in a woman infected by COVID-19 pneumonia. European Heart Journal. 2020 Jun 7;41(22):2133–2133.

14. Khodamoradi Z, Boogar SS, Shirazi FKH, Kouhi P. COVID-19 and Acute Pulmonary Embolism in Postpartum Patient. Emerg Infect Dis. 2020 Aug;26(8):1937–9.

15. Molina MF, Al Saud AA, Al Mulhim AA, Liteplo AS, Shokoohi H. Nitrous oxide inhalant abuse and massive pulmonary embolism in COVID-19. The American Journal of Emergency Medicine. 2020 Jul;38(7):1549.e1-1549.e2.

16. Nauka PC, Oran E, Chekuri S. Deep venous thrombosis in a non-critically ill patient with novel COVID-19 infection. Thrombosis Research. 2020 Aug;192:27–8.

17. Overhoff D, Walter T, Gruettner J, Janssen S, Riffel J, Hoffmann U, et al. Acute pulmonary embolism mimicking COVID – 19 pneumonia. International Journal of Infectious Diseases. 2020 Jul;96:475–6.

18. Poggiali E, Bastoni D, Ioannilli E, Vercelli A, Magnacavallo A. Deep Vein Thrombosis and Pulmonary Embolism: Two Complications of COVID-19 Pneumonia? European Journal of Case Reports in Internal Medicine. 2020 Apr 8;7(5):1.

19. Polat V, Bostancı Gİ. Sudden death due to acute pulmonary embolism in a young woman with COVID-19. J Thromb Thrombolysis. 2020 Jul;50(1):239–41.

20. Qanadli SD, Gudmundsson L, Rotzinger DC. Catheter-directed thrombolysis in COVID-19 pneumonia with acute PE: Thinking beyond the guidelines. Thrombosis Research. 2020 Aug;192:9–11.

21. Schmiady MO. OUP accepted manuscript. European Heart Journal [Internet]. 2020 [cited 2020 Aug 31]; Available from: http://fdslive.oup.com/www.oup.com/pdf/production_in_progress.pdf

22. Tamburello A. COVID-19 and Pulmonary Embolism: Not a Coincidence. European Journal of Case Reports in Internal Medicine. 2020;

23. Ullah W, Saeed R, Sarwar U, Patel R, Fischman DL. COVID-19 complicated by Acute Pulmonary Embolism and Right-Sided Heart Failure. JACC: Case Reports. 2020 Apr;S2666084920303673.

24. Vitali C, Minniti A, Caporali R, Del Papa N. Occurrence of pulmonary embolism in a patient with mild clinical expression of COVID-19. Thrombosis Research. 2020 Aug;192:21–2.

25. Zhou B, She J, Wang Y, Ma X. A Case of Coronavirus Disease 2019 With Concomitant Acute Cerebral Infarction and Deep Vein Thrombosis. Front Neurol. 2020 Apr 22;11:296.

26. Ly A, Alessandri C, Skripkina E, Meffert A, Clariot S, de Roux Q, et al. Rescue fibrinolysis in suspected massive pulmonary embolism during SARS-CoV-2 pandemic. Resuscitation. 2020 Jul;152:86–8.

27. Abernethy K, Sivakumar P, Patrick T, Robbie H, Periselneris J. Coexistent COVID-19 pneumonia and pulmonary embolism: challenges in identifying dual pathology. Thorax. 2020 Sep;75(9):812–4.

28. Cena T, Bazzano S, Berni P, Vignazia GL, Grossi F, Stanca C, et al. Inferior Vena Cava Filter in a Patient with COVID-19 Pneumonia to Prevent a Massive Pulmonary Embolism. Annals of Vascular Surgery. 2020 May;S0890509620304398.

29. Li T, Cheng G, Pipavath SNJ, Kicska GA, Liu L, Kinahan PE, et al. The novel coronavirus disease (COVID‐19) complicated by pulmonary embolism and acute respiratory distress syndrome. J Med Virol. 2020 Jun 12;jmv.26068.

30. Lorenzo C, Francesca B, Francesco P, Elena C, Luca S, Paolo S. Acute pulmonary embolism in COVID-19 related hypercoagulability. J Thromb Thrombolysis. 2020 Jul;50(1):223–6.

31. Tveita A, Hestenes S, Sporastøyl ER, Pettersen SA, Neple BL, Myrstad M, et al. Lungeembolisme ved covid-19. Tidsskriftet [Internet]. 2020 [cited 2020 Aug 31]; Available from: https://tidsskriftet.no/2020/05/kort-kasuistikk/lungeembolisme-ved-covid-19

32. Elavia N, Sharma N, Li S, Wang Y, Milekic B. An Atypical Presentation of Acute Pulmonary Embolism With Severe Acute Respiratory Syndrome Coronavirus 2 (SARS-CoV-2) Pneumonia. Cureus [Internet]. 2020 May 23 [cited 2020 Aug 31]; Available from: https://www.cureus.com/articles/32536-an-atypical-presentation-of-acute-pulmonary-embolism-with-severe-acute-respiratory-syndrome-coronavirus-2-sars-cov-2-pneumonia

33. González del Portillo E, Pérez-Romasanta LA. Pulmonary embolism and acro-ischemia in a lung cancer patient with COVID-19. Medicina Clínica. 2020 Jun;S002577532030350X.

34. Geza Halasz, Francesco Di Spigno, Massimo Piepoli, Giovanni Quinto Villani, Silvia Nardecchia, Tiziana Spezzano, et al. L’embolia polmonare come complicanza tardiva della polmonite da SARS-CoV-2: una serie di casi clinici. Giornale Italiano di Cardiologia [Internet]. 2020 Jul 1 [cited 2020 Aug 31];(2020Luglio). Available from: http://doi.org/10.1714/3386.33638

35. Cavalcanti DD, Raz E, Shapiro M, Dehkharghani S, Yaghi S, Lillemoe K, et al. Cerebral Venous Thrombosis Associated with COVID-19. AJNR Am J Neuroradiol. 2020 Aug;41(8):1370–6.

36. Ali S, Mathew S, Pappachan JM. Acute cor pulmonale from saddle pulmonary embolism in a patient with previous COVID-19: should we prolong prophylactic anticoagulation? International Journal of Infectious Diseases. 2020 Aug;97:299–302.

37. Kariyanna PT, Hossain NA, Jayarangaiah A, Hossain NA, Uppin V, Hegde S, et al. Thrombus in Transit and Impending Pulmonary Embolism Detected on POCUS in a Patient with COVID-19 Pneumonia. Am J Med Case Rep. 2020;8(8):225–8.

38. Gill I, Chan S, Fitzpatrick D. COVID-19-associated pulmonary and cerebral thromboembolic disease. Radiology Case Reports. 2020 Aug;15(8):1242–9.

39. Akel T, Qaqa F, Abuarqoub A, Shamoon F. Pulmonary embolism: A complication of COVID 19 infection. Thrombosis Research. 2020 Sep;193:79–82.

40. Nieri D, Lenzini G, Canari Venturi B, Celi A. Pulmonary embolism: yet another cause of hypoxaemic respiratory failure in COVID-19. ERJ Open Res. 2020 Apr;6(2):00220–2020.

41. Becher Y. D-dimer and C-reactive Protein Blood Levels Over Time Used to Predict Pulmonary Embolism in Two COVID-19 Patients. European Journal of Case Reports in Internal Medicine. 2020;

42. Mahan K, Kabrhel C, Goldsmith AJ. Abdominal pain in a patient with COVID-19 infection: A case of multiple thromboemboli. The American Journal of Emergency Medicine. 2020 May;S0735675720303910.

43. Sung J, Anjum S. Coronavirus Disease 2019 (COVID-19) Infection Associated With Antiphospholipid Antibodies and Four-Extremity Deep Vein thrombosis in a Previously Healthy Female. Cureus [Internet]. 2020 Jun 2 [cited 2020 Aug 31]; Available from: https://www.cureus.com/articles/32973-coronavirus-disease-2019-covid-19-infection-associated-with-antiphospholipid-antibodies-and-four-extremity-deep-vein-thrombosis-in-a-previously-healthy-female

44. Chibane S, Gibeau G, Poulin F, Tessier P, Goulet M, Carrier M, et al. Hyperacute multi-organ thromboembolic storm in COVID-19: a case report. J Thromb Thrombolysis [Internet]. 2020 Jun 6 [cited 2020 Aug 31]; Available from: http://link.springer.com/10.1007/s11239-020-02173-w

45. Motwani M. Abrupt deterioration and pulmonary embolism in COVID-19: a case report. Clin Med. 2020 Jul;20(4):e95–6.

46. Carlsson TL, Walton B, Collin G. Pulmonary artery thrombectomy – a life‐saving treatment in a patient with presumed COVID‐19 complicated by a massive pulmonary embolus. Br J Haematol [Internet]. 2020 Aug [cited 2020 Aug 31];190(3). Available from: https://onlinelibrary.wiley.com/doi/abs/10.1111/bjh.16920

47. Davoodi L, Jafarpour H, Taghavi M, Razavi A. COVID-19 Presented With Deep Vein Thrombosis: An Unusual Presenting. Journal of Investigative Medicine High Impact Case Reports. 2020 Jan;8:232470962093123.

48. Moreira BL, Santana PRP, Zanetti G, Marchiori E. COVID-19 and acute pulmonary embolism: what should be considered to indicate a computed tomography pulmonary angiography scan? Rev Soc Bras Med Trop. 2020;53:e20200267.

49. Hékimian G, Lebreton G, Bréchot N, Luyt C-E, Schmidt M, Combes A. Severe pulmonary embolism in COVID-19 patients: a call for increased awareness. Crit Care. 2020 Dec;24(1):274.

50. Bellieni A, Intini E, Taddei E, Baldi F, Larosa L, Murri R, et al. Challenges in COVID-19: is pulmonary thromboembolism related to overall severity? Infectious Diseases. 2020 Aug 2;52(8):585–9.

51. Vailati D, Fusco T, Canelli A, Chiariello C, Zerla P. Axillary vein thrombosis in COVID positive patient with midline and continuous positive airway pressure Helmet. J Vasc Access. 2020 Jul 15;112972982094342.

52. Lingamaneni P, Gonakoti S, Moturi K, Vohra I, Zia M. Heparin-Induced Thrombocytopenia in COVID-19. Journal of Investigative Medicine High Impact Case Reports. 2020 Jan;8:232470962094409.

53. Martin AI, Rao G. COVID-19: A Potential Risk Factor for Acute Pulmonary Embolism. Methodist Debakey Cardiovasc J. 2020 Jun;16(2):155–7.

54. Casey K, Iteen A, Nicolini R, Auten J. COVID-19 pneumonia with hemoptysis: Acute segmental pulmonary emboli associated with novel coronavirus infection. The American Journal of Emergency Medicine. 2020 Jul;38(7):1544.e1-1544.e3.

55. Martinelli I, Ferrazzi E, Ciavarella A, Erra R, Iurlaro E, Ossola M, et al. Pulmonary embolism in a young pregnant woman with COVID-19. Thrombosis Research. 2020 Jul;191:36–7.

56. Bozzani A, Arici V, Franciscone MM, Danesino V, Cascina A, Ticozzelli G, et al. Severe Acute Respiratory Syndrome Coronavirus 2 Infection and the Upper Limb Deep Vein Thrombosis Risk. Annals of Vascular Surgery. 2020 Jul;66:11–3.

57. Sulemane S, Baltabaeva A, Barron AJ, Chester R, Rahman-Haley S. Acute pulmonary embolism in conjunction with intramural right ventricular thrombus in a SARS-CoV-2-positive patient. European Heart Journal - Cardiovascular Imaging. 2020 Sep 1;21(9):1054–1054.

58. Marsico S, Espallargas Giménez I, Carbullanca Toledo SJ, Del Carpio Bellido LA, Maiques Llácer JM, Zuccarino F. Pulmonary infarction secondary to pulmonary thromboembolism in COVID-19 diagnosed with dual-energy CT pulmonary angiography. Revista Española de Cardiología (English Edition). 2020 Aug;73(8):672–4.

59. Hughes C. Cerebral Venous Sinus Thrombosis as a Presentation of COVID-19. European Journal of Case Reports in Internal Medicine. 2020;

60. Brüggemann R, Gietema H, Jallah B, ten Cate H, Stehouwer C, Spaetgens B. Arterial and venous thromboembolic disease in a patient with COVID-19: A case report. Thrombosis Research. 2020 Jul;191:153–5.

61. Dahl-Cruz F. Trombosis venosa cerebral e infección por SARS-CoV-2. Rev Neurol. 2020;

62. Pishgahi M, Ansari Aval Z, Hajimoradi B, Bozorgmehr R, Safari S, Yousefifard M. Massive Pulmonary Thromboembolism in Patients with COVID-19; Report of Three Cases. Arch Acad Emerg Med. 2020;8(1):e58.

63. Stoneham SM, Milne KM, Nuttall E, Frew GH, Sturrock BR, Sivaloganathan H, et al. Thrombotic risk in COVID-19: a case series and case–control study. Clin Med. 2020 Jul;20(4):e76–81.

64. Klein DE, Libman R, Kirsch C, Arora R. Cerebral venous thrombosis: A typical presentation of COVID-19 in the young. Journal of Stroke and Cerebrovascular Diseases. 2020 Aug;29(8):104989.

65. Lal S. A Rare Finding of Upper Limb Deep Venous Thrombosis in a Patient with COVID-19. J Coll Physicians Surg Pak. 2020 Jun 1;30(1):76–7.

66. Ahmed T, Ahmed T, Kumar S, Lodhi SH, Akbik B. Rare Presentation of Pulmonary Embolism Amidst Coronavirus Disease 2019 Era: Utility of Multiorgan Ultrasonography. Cureus [Internet]. 2020 Jun 5 [cited 2020 Aug 31]; Available from: https://www.cureus.com/articles/33384-rare-presentation-of-pulmonary-embolism-amidst-coronavirus-disease-2019-era-utility-of-multiorgan-ultrasonography

67. Elkattawy S, Younes I, Noori MAM. A Case Report of Polymerase Chain Reaction-Confirmed COVID-19 in a Patient With Right Ventricular Thrombus and Bilateral Deep Vein Thrombosis. Cureus [Internet]. 2020 Jun 15 [cited 2020 Aug 31]; Available from: https://www.cureus.com/articles/33382-a-case-report-of-polymerase-chain-reaction-confirmed-covid-19-in-a-patient-with-right-ventricular-thrombus-and-bilateral-deep-vein-thrombosis

68. Di Tano G. Late Pulmonary Embolism after COVID-19 Pneumonia despite Adequate Rivaroxaban Treatment. European Journal of Case Reports in Internal Medicine. 2020;

69. Collange O, Tacquard C, Delabranche X, Leonard-Lorant I, Ohana M, Onea M, et al. Coronavirus Disease 2019: Associated Multiple Organ Damage. Open Forum Infectious Diseases. 2020 Jul 1;7(7):ofaa249.

70. Riker RR, May TL, Fraser GL, Gagnon DJ, Bandara M, Zemrak WR, et al. Heparin‐induced thrombocytopenia with thrombosis in COVID‐19 adult respiratory distress syndrome. Res Pract Thromb Haemost. 2020 Jul;4(5):936–41.

71. Garg A, Goyal S, Patel P. A Case of COVID-19 Infection With Delayed Thromboembolic Complication on Warfarin. Cureus [Internet]. 2020 Jun 26 [cited 2020 Aug 31]; Available from: https://www.cureus.com/articles/35187-a-case-of-covid-19-infection-with-delayed-thromboembolic-complication-on-warfarin

72. Baudar C, Duprez T, Kassab A, Miller N, Rutgers MP. COVID-19 as triggering co-factor for cortical cerebral venous thrombosis? Journal of Neuroradiology. 2020 Jun;S0150986120302054.

73. Logan G. Hypercoagulable State in COVID-19: A Case Series of Three Patients. Cureus. 2020;

74. Rigamonti A, Mantero V, Piamarta F, Spena G, Salmaggi A. Cerebral venous thrombosis associated with coronavirus infection: an underestimated entity? Neurol Sci [Internet]. 2020 Jun 29 [cited 2020 Aug 31]; Available from: http://link.springer.com/10.1007/s10072-020-04539-7

75. Ataallah B, Sharma A, Tamanna S, Ng J, Haggerty G. Major Thrombotic Event Despite Anticoagulation in a Patient With COVID-19. Cureus [Internet]. 2020 Jun 29 [cited 2020 Aug 31]; Available from: https://www.cureus.com/articles/34821-major-thrombotic-event-despite-anticoagulation-in-a-patient-with-covid-19

76. Fortuzi K, Ghazanfar H, Haider A, Patel K, Patel M. Pulmonary Embolism in COVID-19 Pneumonia: Random Association or Causality? Cureus [Internet]. 2020 Jun 29 [cited 2020 Aug 31]; Available from: https://www.cureus.com/articles/35250-pulmonary-embolism-in-covid-19-pneumonia-random-association-or-causality

77. Patil NR, Herc ES, Girgis M. Cold agglutinin disease and autoimmune hemolytic anemia with pulmonary embolism as a presentation of COVID-19 infection. Hematology/Oncology and Stem Cell Therapy. 2020 Jul;S1658387620301163.

78. Argirò R, Cirelli C, Sgreccia A, Del Sette B, Da Ros V. Cerebral hemorrhage related to vein thrombosis in Covid-19 patients in different Italian hospitals: View point for clinical and imaging implications. Journal of the Neurological Sciences. 2020 Sep;416:117023.

79. Roy-Gash F, De Mesmay M, Devys J-M, Vespignani H, Blanc R, Engrand N. COVID-19-associated acute cerebral venous thrombosis: clinical, CT, MRI and EEG features. Crit Care. 2020 Dec;24(1):419.

80. Visveswaran GK, Morparia K, Narang S, Sturt C, Divita M, Voigt B, et al. Severe Acute Respiratory Syndrome Coronavirus 2 Infection and Thrombosis: Phlegmasia Cerulea Dolens Presenting with Venous Gangrene in a Child. The Journal of Pediatrics. 2020 Jul;S0022347620308751.

81. Della Bona R, Valbusa A, La Malfa G, Giacobbe DR, Ameri P, Patroniti N, et al. Systemic fibrinolysis for acute pulmonary embolism complicating acute respiratory distress syndrome in severe COVID-19: a case series. European Heart Journal - Cardiovascular Pharmacotherapy. 2020 Jul 14;pvaa087.

82. Akiyama Y, Horiuchi K, Kondo Y, Kabata H, Ishii M, Fukunaga K. A case of non‐severe COVID ‐19 complicated by pulmonary embolism. Respirology Case Reports [Internet]. 2020 Oct [cited 2020 Aug 31];8(7). Available from: https://onlinelibrary.wiley.com/doi/abs/10.1002/rcr2.622

83. Fulgencio-Barbarin J, Calleja-Algarra A, Morales-Raya C. Trombosis sistémica producida por COVID-19. Medicina Clínica. 2020 Jul;S0025775320303523.

84. Shah K. SARS-CoV-2 and Thrombosis: More Than Just by Chance. Am J Med Sci. 2020 Jul 17;

85. Haider MB. A 46-Year-Old Woman Who Presented with Diabetic Ketoacidosis and COVID-19 Pneumonia with Multiple Pulmonary Thromboemboli: A Case Report. Am J Case Rep. 2020;

86. Borghesi A, Aggiusti C, Farina D, Maroldi R, Muiesan ML. COVID-19 Pneumonia: Three Thoracic Complications in the Same Patient. Diagnostics. 2020 Jul 20;10(7):498.

87. Alharthy A, Faqihi F, Papanikolaou J, Balhamar A, Blaivas M, Memish ZA, et al. Thrombolysis in severe COVID-19 pneumonia with massive pulmonary embolism. The American Journal of Emergency Medicine. 2020 Jul;S073567572030663X.

88. Fujikura K, Fontes JD, Taub CC. Saddle pulmonary embolism and thrombus‐in‐transit straddling the patent foramen ovale 28 days after COVID symptom onset. Echocardiography. 2020 Jul 31;echo.14796.

89. Arachchillage DJ, Stacey A, Akor F, Scotz M, Laffan M. Thrombolysis restores perfusion in COVID‐19 hypoxia. Br J Haematol. 2020 Aug 17;bjh.17050.
